# Comparison of the Antioxidant Activities and Polysaccharide Characterization of Fresh and Dry *Dendrobium officinale Kimura et Migo*

**DOI:** 10.3390/molecules27196654

**Published:** 2022-10-07

**Authors:** Wang Zhang, Xinjie Liu, Xun Sun, Rongchun Han, Nianjun Yu, Juan Liang, An Zhou

**Affiliations:** 1 The Experimental Research Center, Anhui University of Chinese Medicine, Hefei 230038, China; 2 School of Pharmacy, Anhui University of Chinese Medicine, Hefei 230013, China; 3 Anhui Province Key Laboratory of Research and Development of Chinese Medicine, Anhui Province Key Laboratory of Chinese Medicinal Formula, Hefei 230012, China

**Keywords:** *Dendrobium officinale*, antioxidant activity, polysaccharide, mannose, monosaccharide composition

## Abstract

It is generally believed that fresh *Dendrobium officinale* (FDO) has more significant pharmacological activity than dried *Dendrobium officinale* (DDO); however, the difference has not been clearly shown. Our study compared their antioxidant properties both in vitro and in vivo, and the molecular weight arrangement and monosaccharide composition of the fresh *Dendrobium officinale* polysaccharides (FDOPs) and the dried *Dendrobium officinale* polysaccharides (DDOPs) were analyzed by HPLC-GPC and GC-MS. The results showed that the FDO and its polysaccharides had more significant effects on scavenging DPPH, ABTS, and hydroxyl radicals than the DDO. In addition, both the FDO and DDO significantly reduced lipid peroxidation levels and increased the SOD, T-AOC, CAT, and GSH levels in mice with acute liver damage caused by CCl_4_, while the FDO and its polysaccharides were more effective. Histopathological analysis further verified the protective effect of the Dendrobium polysaccharides on CCl_4_-induced liver injury. The determination of the polysaccharides revealed that the polysaccharide and mannose contents of the FDO were significantly higher than their dried counterparts, and the homogeneous arrangement of the polysaccharides in the FDO was degraded into three polysaccharide fragments of different molecular weights in the DDO. Overall, our data identified differences in the antioxidant activities of the FDO and DDO, as well as the reasons for these differences.

## 1. Introduction

Fresh medicinal herbs, traditional Chinese folk medicine, have been used in China to treat a variety of ailments for thousands of years. It is generally accepted that fresh medicinal herbs possess higher pharmacological activities than their dried forms [1,2]. Additionally, the content of the active ingredients of fresh herbs is much higher than their dried forms, which undergo a series of physical and chemical processes. The drying process may lead to the degradation of some of the chemical components of fresh herbs, especially heat-sensitive compounds, such as polyphenols, phenolic acids, and volatile oils [3,4,5], etc. Compared with dried *Portulaca oleracea L.* (POL) extracts, the fresh extract showed stronger antidiabetic activity and the relative contents of polyphenols and alkaloids were more abundant [6]. Similarly, the α- turmerone and β-turmerone contents of dried rhizomes of turmeric were significantly lower than that of fresh turmeric, and the antioxidant properties were significantly impaired [7]. On the contrary, the heat-treating process may increase the content of the compounds or the pharmacological capacities of some herbs, for example, polyphenols in lemon and dried Citrus peels [8,9] and antioxidative activities in onion [10]. Thus, whether there are differences in the chemical compositions or pharmacological properties of fresh *Dendrobium officinale* (FDO) and dried *Dendrobium officinale* (DDO) is worth exploring.

In many Asian countries, the dried stem of *Dendrobium officinale Kimura et Migo* (DO) has been widely used as a herbal medicine for thousands of years [11]. About 1300 years ago, DO was reported as “one of the most famous Dendrobium in China”, due to its health benefits during the Tang dynasty [12]. As a rare and remotely noted traditional Chinese medicine, DO has unparalleled medicinal value and clinical use, mainly prescribed to promote the production of body fluid, nourish the stomach and body, clear away heat-evil, moisten the lungs, etc. [13]. The polysaccharides are the most abundant bioactive components in DO, reaching 35%, and mainly composed of d-mannose and d-glucose, with the highest proportion of mannose [14,15,16]. The *Dendrobium officinale* polysaccharides (DOPs) exert antioxidant effects by scavenging free radicals and enhancing the antioxidant defense system of the body [17]. Therefore, the antioxidant activity of DO is closely correlated to the content and chemical properties of polysaccharides [18]. The other active ingredients of DO, including flavonoids, alkaloids, bibenzyls and phenanthrenes, which have pharmacological activities such as antioxidant, immune, antitumor, and hypoglycemic [19,20,21,22,23], with very little content [24]. Therefore, we mainly focused on the polysaccharide content and monosaccharide composition of DO.

Dendrobium has been squeezed into fresh juice, owing to its original natural efficiency. However, since it is difficult to store the fresh herb, it has been mainly replaced by dried forms in the marketplace. Studies have demonstrated that fresh Dendrobium is superior to the dried form in its pharmacological activity. Accumulating evidence suggests that the content of the active components of the fresh herbs, which may prevent and treat some diseases, is much higher than the dried form [25,26]. In consideration of the differences in chemical constituents and pharmacological activities of fresh and dried herbs, there is currently a lack of research on the differences in antioxidant activities between FDO and DDO, especially the relationships between the antioxidant activities of DO and the changes in its active ingredients.

The aim of this study was to elucidate the differences in the antioxidant activities of the FDO and DDO and to explain them in terms of the polysaccharide content and composition, providing a theoretical basis for the development and utilization of FDO.

## 2. Results

### 2.1. The Antioxidant Activity of the FDO and DDO In Vitro

**Figure 1:**
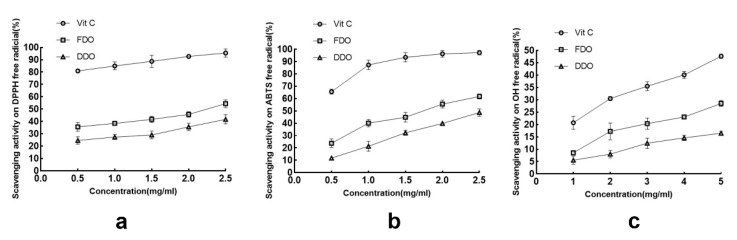
The scavenging effects of the FDO (fresh *Dendrobium officinale*) and DDO (dried *Dendrobium officinale*) on DPPH (**a**), ABTS (**b**), and hydroxyl free radicals (**c**).

The scavenging activities of the DPPH radicals, ABTS radicals, and hydroxyl radicals were measured for the diluted FDO and DDO samples (Figure 1). With the increase in the concentration of the three samples, the DPPH radical scavenging capacities were gradually enhanced. Undoubtedly, the scavenging activity of Vitamin C was higher than the FDO and DDO (*p* < 0.01), containing the highest elimination rate by 95% and 98 μg/mL of IC_50_. It was noteworthy that FDO at 2.5 mg/mL had the highest DPPH scavenging rate of 52.62% with an IC_50_ of 2.624 mg/mL. The FDO was significantly better than the DDO (*p* < 0.05), which was 39.34% at the same concentration, and the IC_50_ was 3.584 mg/mL. Both the FDO and DDO samples showed a strong ability to scavenge ABTS radicals. The abilities were increased with higher sample concentrations, within the test dose range. At the concentration of 0.5–2.5 mg/mL, the ability of the FDO to scavenge the ABTS radicals was measured to be 23.12–61.24% (IC_50_ = 1.68 mg/mL), while the DDO sample was assayed to be 11.7–48.01% (IC_50_ = 2.762 mg/mL), which was lower than fresh samples (*p* < 0.05). As with the above results, although the FDO and DDO’s scavenging of hydroxyl radicals increased with the increase in their concentration, the fact that the IC_50_ value of the FDO (IC_50_ = 15.975 mg/mL) was lower than that of the DDO (IC_50_ = 42.267 mg/mL) suggested that the scavenging effect of the FDO was superior to that of the DDO (*p* < 0.05).

### 2.2. The Antioxidant Activity of the FDO and DDO In Vivo

#### 2.2.1. The Effect of the FDO and DDO on Antioxidative Parameters in the Liver and Serum

**Figure 2:**
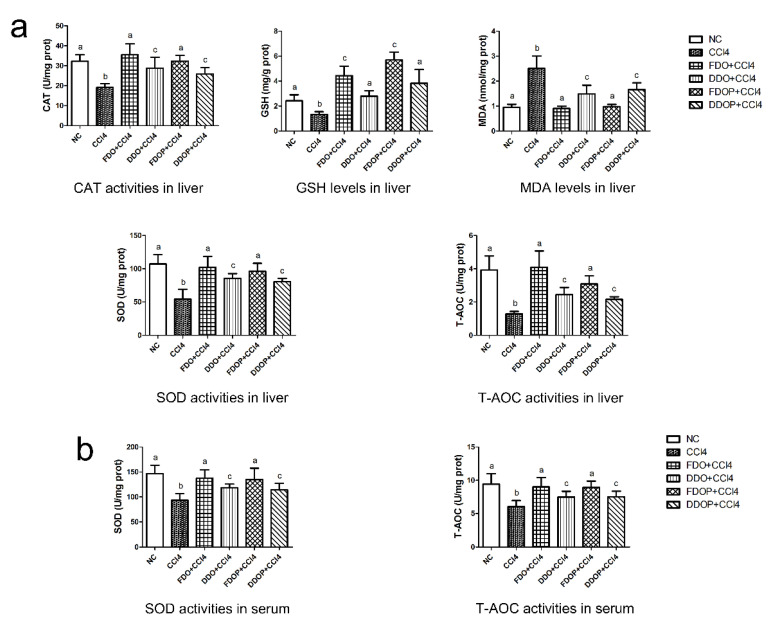
Effects of FDO, DDO, FDOP (fresh *Dendrobium officinale* polysaccharide), and DDOP (dried *Dendrobium officinale* polysaccharide) on antioxidant parameters including CAT activities, GSH levels, MDA levels, SOD activities, and T-AOC activities in the liver, (**a**) and SOD and T-AOC) activities in serum (**b**) of CCl_4_-treated mice. All data were presented as means ± SD (*n* = 6) and analyzed with one-way analysis of variance (ANOVA) followed by least significant differences (LSD) multiple comparison and Dunnett’s T3 tests. Different alphabets (a–c) in pictures showed a significant difference (*p* < 0.05). NC: negative control; CCl_4_, tetrachloromethane, widely used to induce toxic liver injury in various experimental animals.

The antioxidant properties of the FDO and DDO were further confirmed by in vivo determination. Oxidative stress biomarkers, such as the T-AOC, GSH, CAT, SOD, and MDA, were measured in the liver and serum (Figure 2). The levels of T-AOC, GSH, CAT, and SOD were significantly decreased, whereas the MDA level was increased in the CCl_4_-induced toxic control, compared with the normal control, FDO, and DDO groups (*p* < 0.05). In addition, the FDO groups showed the maximum increase in the liver and serum of the levels of T-AOC, GSH, CAT, and SOD and an obvious decrease in the MDA level with the same treatment, compared to the DDO groups (*p* < 0.05). The findings indicated that the FDO had stronger antioxidative activity than the DDO on the CCl_4_-induced toxic mice.

#### 2.2.2. The Effect of the FDO and DDO on the Histopathology

**Figure 3:**
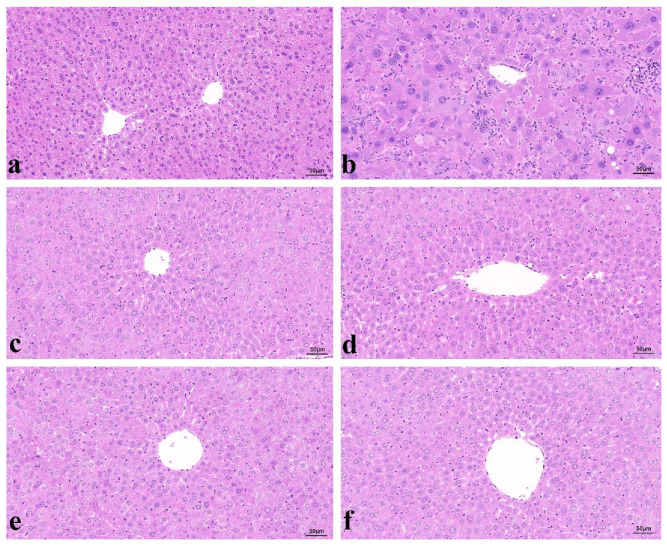
Histopathological effects of FDO, DDO, FDOP, and DDOP on CCl_4_-induced hepatic injury (×200). Liver tissues were obtained from normal control mice (**a**), CCl_4_-treated mice (**b**), FDO + CCl_4_-treated mice (**c**), DDO + CCl_4_-treated mice (**d**), FDOP + CCl_4_-treated mice (**e**), DDOP + CCl_4_-treated mice (**f**). NC: negative control; CCl_4_, tetrachloromethane, widely used to induce toxic liver injury in various experimental animals.

To study the inhibitory effects of FDO and DDO administration on CCl_4_-induced toxic mice, the histopathological changes in liver tissue with H&E staining were observed by microscope. The normal control mice had a cellular structure of normal hepatocytes and the central hepatic vein (Figure 3a). In contrast, a deteriorated hepatic architecture with partial inflammation of hepatocytes was presented in the liver of the CCl_4_-induced toxic group (Figure 3b). In particular, the maximum protection was discovered in the FDO group (Figure 3c). The FDO group showed mild histopathological changes, compared to the DDO group, which also had limited protection (Figure 3d). These results coincided well with the biochemical analysis.

### 2.3. The Content of Polysaccharides in the FDO and DDO

Many studies have demonstrated that the polysaccharides in Dendrobium are closely related to the pharmacological activity [27,28]. Following the extraction mentioned above, the contents of the polysaccharides were determined according to the phenol–sulfuric acid method (Table 1). In the FDO samples, the contents of polysaccharides were 512.7 ± 13.32 mg/g of extract and significantly higher than in the DDO samples (*p* < 0.01), which were 348.7 ± 12.82 mg/g of extract, suggesting that drying may affect the polysaccharides in DO.

### 2.4. The Content of Mannose in the FDO and DDO

The mannose, obtained from the extraction, hydrolyzation, and derivatization with PMP, was analyzed by HPLC (Table 1), and the results showed that the content of mannose in the FDO was 489.5 ± 2.757 mg/g of the total amount, but the mannose content in the DDO was only 291.3 ± 5.572 mg/g of the total weight. In the DDO, the mannose content, which was markedly less (*p* < 0.05) than the FDO, may be due to the absence of polysaccharides, which are the source of mannose.

### 2.5. The Antioxidant Activity of the FDOP and DDOP In Vivo

#### 2.5.1. The Effect of the FDOP and DDOP on the Antioxidative Parameters in the Liver and Serum

To investigate the differences of the FDOP and DDOP on antioxidant activities, we examined the levels of the T-AOC, GSH, CAT, SOD, and MDA in the liver and serum of CCl_4_-induced intoxicated mice (Figure 2). Obviously, the CCl_4_-induced toxic group had significantly decreased T-AOC, GSH, CAT, and SOD levels and an increased MDA level in the liver and serum, compared with the normal control, FDOP, and DDOP groups (*p* < 0.05). Both the FDOP and DDOP groups showed antioxidant activity; however, the FDOP was much higher than the DDOP group (*p* < 0.05). For this study, the result could be interpreted as drying leading to changes in the molecular weight and structure of the polysaccharides in the DDO.

#### 2.5.2. The Effect of the FDOP and DDOP on the Histopathology

In the same way, to verify the biochemical results obtained from the liver and serum, the histopathology of the liver tissue was studied again (Figure 3). The liver histopathology of the normal control animals showed a typical structure of a hepatocyte accompanied with a central vein, opposite to the CCl_4_-induced toxic mice, which exhibited hepatocyte architecture distortion of the central vein in the liver tissue. Furthermore, the FDOP group was observably better than the DDOP group at protecting the liver tissue from CCl_4_-induced toxicity (Figure 3e,f). In addition, it was verified that the FDOP was different to the DDOP in its bioactivity.

### 2.6. Molecular Weight Analysis and the Monosaccharide Composition of the FDOP and DDOP

**Figure 4:**
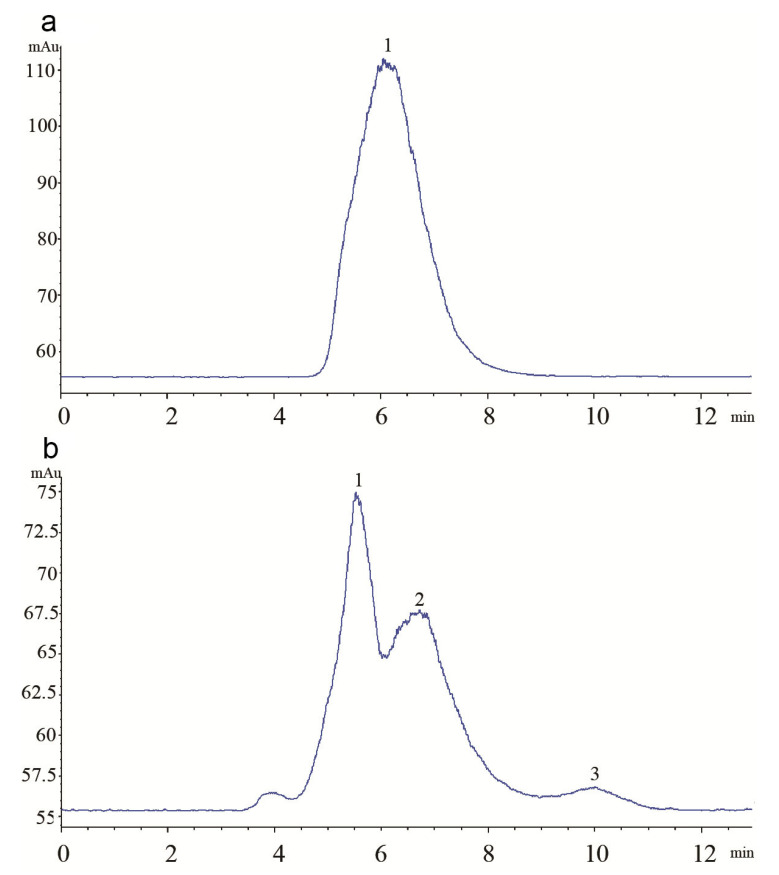
The molecular weight analysis of the FDOP (**a**) and DDOP (**b**) was shown by HPLC-GPC.

To find the differences in the effect of the FDOP and DDOP on the chemical compositions, their molecular mass compositions were determined by HPLC-GPC. One chromatographic peak of polysaccharide macromolecules was shown in the FDOP, and three different chromatographic peaks were shown in the DDOP, which contained one peak of polysaccharide macromolecules and two small polysaccharide peaks (Figure 4). Thus, it was easy to find polysaccharides in an intact form in the FDO, but the polysaccharides were broken into three different segments of the molecular weight by drying the DDO.

**Figure 5:**
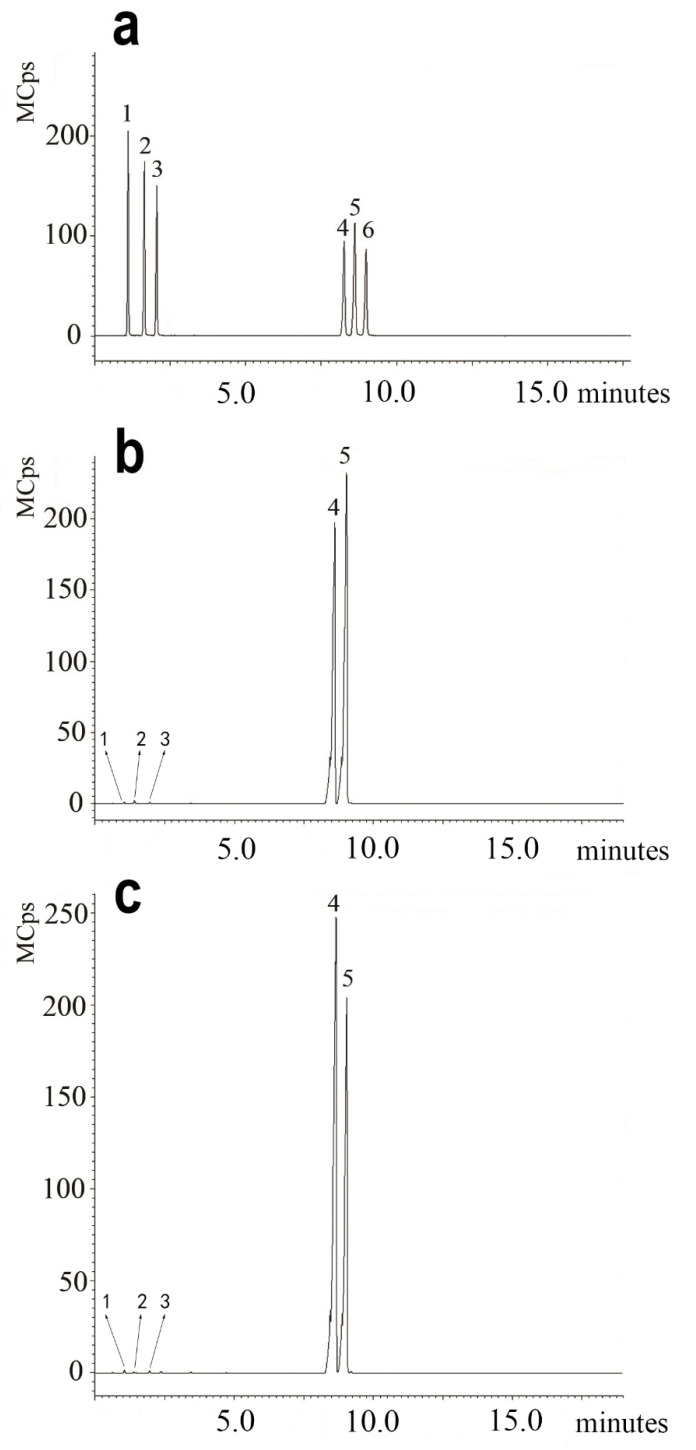
Monosaccharide standard and monosaccharide components of the FDOP and DDOP were analyzed by GC-MS. Mixed standard solutions of monosaccharides (**a**) (rhamnose, aldose, xylose, mannose, glucose and galactose in the sequence from left to right 1–6); FDOP samples (**b**); DDOP samples (**c**).

The monosaccharide composition of the FDOP and DDOP was analyzed by GC-MS (Figure 5). There was a noticeable difference between the FDOP and DDOP in terms of the monosaccharide composition (Table 2). When the relative content of glucose was 1, the relative content of mannose was 0.830, and the relative content of rhamnose was 0.008 in the FDOP. However, the relative contents of glucose, mannose, and rhamnose were, respectively, 0.719, 1.000, and 0.001 in the DDOP. The ingredients of the monosaccharides were mainly glucose, mannose, rhamnose, and galactose in the FDOP and DDOP, and there were differences in the relative abundance of glucose, mannose, and rhamnose between the FDOP and DDOP. These differences may be important reasons why the FDOP showed more antioxidant activity than the DDOP.

## 3. Discussion

In terms of in vitro antioxidant activity, the models of free radicals including DPPH, ABTS, and hydroxyl are widely used for antioxidant testing. The DPPH molecule is a stable radical with maximum absorbance at 517 nm, used for quickly assessing the capacity of antioxidants [29]. The ABTS test is frequently used in measuring the total antioxidant ability of a single compound mixed with various samples in organic or aqueous solvent systems [30]. The hydroxyl free radical is the most ROS and generates oxidative injury to biomolecules by a series of reactions [31]. In this study, both the FDO and DDO performed well in scavenging the DPPH, ABTS, and hydroxyl radicals (Figure 1). However, the IC_50_ of scavenging in the FDO was significantly less than in the DDO, indicating that the FDO had higher antioxidant properties compared with the DDO.

CCl_4_, a well-known hepatotoxin, is widely used to induce toxic liver injury in various laboratory animals [32]. In CCl_4_-induced toxicity, the pathogenesis is that CCl_4_ produces excessive free radicals containing trichloromethyl radicals (CCl_3_) and trichloromethyl peroxyl radicals (CCl_3_O_2_) through the cytochrome P450 system [33]. Those radicals quickly bond to cellular macromolecules, such as lipids and proteins, which eventually causes lipid peroxidation and interference with the systemic redox balance [34]. Then, the antioxidative enzymes including SOD, GSH, and CAT firstly defend against ROS in the organism and turn the active oxygen molecules into nontoxic compounds in the liver. The levels of SOD, GSH, and CAT then decrease due to the gradual accumulation of superoxide and hydrogen peroxide free radicals [35]. MDA, a biomarker of lipid peroxidation, increases, suggesting that more lipid peroxidation is generated by CCl_4_ [36]. The T-AOC values are reported as the statement of the nonenzymatic antioxidant ability in the tissues [37]. In this study, at the same dose level (2 g/kg), the FDO caused a significant increase in the T-AOC, SOD, GSH, and CAT levels and a decrease in the MDA level in the liver and serum of CCl_4_-induced toxic mice compared to the DDO (Figure 2), suggesting that the FDO is superior to the DDO in its antioxidant capacity. The FDOP also had high levels of T-AOC, SOD, GSH, and CAT and low levels of MDA compared to the DDOP, suggesting that the FDOP was significantly stronger than the DDOP.

In this research, the differences in the antioxidant effects of the FDO and DDO were further determined by histopathological examinations with H&E staining. The histopathological examination of liver sections proved that the normal hepatocyte architecture was damaged in the CCl_4_-induced toxic mice (Figure 3a). The liver sections from the FDO and DDO treated samples showed that they both played a role in preventing hepatocytes from CCl_4_-induced toxicity, and the FDO showed a significantly better protective effect than the DDO (Figure 3c,d). In addition, this histopathological finding was consistent with the antioxidant results.

The active ingredients are vital to the antioxidant capacity of DO, particularly polysaccharide, which is the main component contributing to the oxidation resistance of DO [38]. This study showed that the contents of polysaccharide and mannose in the FDO were significantly higher than in the DDO (Table 1), which could be due to the loss of polysaccharides caused by drying. In addition, the effects of the FDOP were observably stronger than the DOP in enhancing the antioxidant activities in vivo and protecting hepatocytes from CCl_4_-induced toxicity in the liver histopathology (Figure 3e,f). The determination of the molecular weights of the FDOP and DDOP using HPLC-GPC revealed that the FDOP had one peak of polysaccharides, while the DDOP was decomposed into three different molecular weight fragments (Figure 4). This finding suggested that the polysaccharide macromolecules in the FDO were probably decomposed into small molecular polysaccharide segments by the drying process in DDO, which further influenced its intrinsic effectiveness.

It was shown that traditional processing would change the contents of the key metabolites and improve the biological activities of DO [39]. Therefore, we further investigated the differences in monosaccharide composition between the FDOP and DDOP (Table 2). The monosaccharide compositions were mainly glucose and mannose, but the relative content of both was different in the FDOP and DDOP (Figure 5). In addition, the relative content of rhamnose in the FDOP was higher than in the DDOP. The FDOP was different from the DDOP in its monosaccharide composition, which might be another reason why the FDO was superior to the DDO in its antioxidant activity.

Today, an increasing number of people are paying more attention to fresh herbs, which are very different from dry ones in their pharmacological effects and chemical compositions. In general, fresh herbs are superior to processed ones in their bioactivities and effective ingredients [1]. DO is known for its antioxidant activity. Comparing the antioxidant activities of FDO and DDO, it was found that there was a considerable loss of antioxidant properties during the drying, implying a reduction in the beneficial pharmacological activities of DO. In this study, the higher antioxidant activities of the FDO were due to the polysaccharides with advantages in content and pharmacological activities, as compared to the DDO. The reason could be that, during the drying process, the polysaccharides and their bioactivities are reduced. The study provided a theoretical basis for the priority of FDO in clinical treatment.

## 4. Materials and Methods

### 4.1. Reagents

Methanol, ethanol, and acetonitrile were LC grade from Merck Chemical Co. (Darmstadt, Germany). In addition, 1,1-diphenyl-2-picrylhydrazyl (DPPH), 1,10-phenanthroline monohydrate, and 2,2-azinobis (3-ethyl-benzothiazoline-6-sulfonic acid) (ABTS) were purchased from Aladdin Biological Technology Co. (Shanghai, China). The total antioxidant capacity (T-AOC) kit, glutathione (GSH) kit, catalase (CAT) kit, malondialdehyde (MDA) kit, superoxide dismutase (SOD) kit, and the Coomassie brilliant blue protein assay kit were obtained from the Nanjing Jiancheng Bioengineering Institute (Nanjing, China). The standards of glucose, galactose, arabinose, D-mannose, rhamnose, and xylose were purchased from Sigma Chemical Co. (St. Louis, MI, USA). All other chemicals and solvents were of analytical grade and obtained from standard commercial suppliers. The DHG-9053A electric constant temperature blast dryer was from Shanghai Medical Constant Temperature Equipment Factory (Shanghai, China); the RE-3000 rotary evaporator and KQ-500DB CNC ultrasonic cleaner were from Kunshan Ultrasonic Instruments Co. (Kunshan, China); the UV-2550 UV-Visible spectrophotometer was purchased from Shimadzu (Tokyo, Japan). The ALPHA1-2LD vacuum freeze dryer was from Martin Christ (Osterode, Germany); an SHB-IIIA circulating water multi-purpose vacuum pump was from Zhengzhou Greatwall Scientific and Industrial Trade Co. (Zhengzhou, China); the CP225D one hundred thousandth electronic balance was from Sartorius (Goettingen, Germany); and the MULTISKAN FC enzyme labeler and ST-16R high-speed frozen centrifuge were from Thermo (Waltham, MA, USA). GC-MS was from Bruker (Karlsruhe, Germany).

### 4.2. Plant Materials

The fresh stems of DO were collected in Anhui province, China, and identified by Nianjun Yu of the Anhui University of Chinese Medicine as the fresh stems of *Dendrobium officinale kimura et Migo*, a perennial epiphytic herb of *Dendrobium officinale* of *Orchidaceae*. In total, 500 g of the fresh stems of DO (FDO) were washed with double-distilled water, drained, cut into small 0.5 cm pieces, and divided into two groups equally. One was the fresh group and stored at −4 °C for the preparation of the FDO and FDOP sample solutions. The other group was dried at 65 °C for 48 h in a thermostatic drum wind drying oven, then ground into 70 screen mesh granules, and stored in desiccator for the preparation of the DDO and DDOP samples. The determination of antioxidant activity and polysaccharide content and composition in dried and fresh DO was completed within 4 weeks. Both the FDO and DDO were subjected to the same analysis. The analytical weights of the fresh and dried samples were equal and considered the moisture loss of the material during storage and drying. This allowed the comparison of results expressed as per dried matter. Six 100 g fresh stems of DO were dried to a constant weight to obtain 22.53 g, 18.16 g, 21.32 g, 19.56 g, 20.90 g, and 21.19 g of dried DO, respectively. After calculation, the average water content of the fresh DO was 79.525%.

The polysaccharides of the fresh DO (FDOP) and the polysaccharides of the dried DO (DDOP) were obtained using the water-extraction and ethanol-precipitation methods, respectively.

### 4.3. Determination of the Antioxidant Activities of the FDO and DDO In Vitro

#### 4.3.1. Sample Preparation

The FDO and DDO were mixed with distilled water and extracted by ultrasound at 50 °C for 1.5 h. After filtration, the residues were re-ultrasounded for 1 h in the same conditions. The combined filtrates were centrifuged at 1500 rpm for 10 min. Then, the precipitates were discarded to obtain the supernatant at a final volume of 200 mL; its concentration was 5 mg/mL. The equivalent dry weight 1.0 g FDO and DDO were mixed with 100 mL distilled water and extracted by ultrasound at 50 °C for 1.5 h.

#### 4.3.2. DPPH Radical Scavenging Activity Assay

One of the antioxidant activities of the samples was analyzed by investigating their ability to scavenge the DPPH free radical, adopting the method described previously [40] with some modifications. First, 1 mL of a 0.1 mM DPPH methanol solution was diluted to different concentrations (2.5, 2.0, 1.5, 1.0, and 0.5 mg/mL) as a test; 0.1 mM DPPH methanol solution served as the control. Methanol was used as the blank solution. After 30 min of reaction at room temperature in the dark, the decrease in the absorbance of the DPPH free radical was measured using a UV-2250 spectrophotometer at 517 nm against a blank. Vitamin C was used as the reference standard. All tests were run in triplicate to calculate the mean values. The DPPH radical scavenging effect was calculated by the following formula:

Scavenging (%) = (A_control_ − A_test_)/A_control_ × 100

#### 4.3.3. ABTS Radical Scavenging Activity Assay

The activity of scavenging the ABTS radical was determined according to the method of the ABTS radical cation (ABTS+) test as described previously [41] with slight modifications. The ABTS solution (7 mM) was mixed with 140 mM of potassium persulphate, and the mixture was stood in the dark at room temperature for 16 h to create a stable ABTS+. Upon assaying, the solution was then diluted with double-distilled water to an absorbance of 0.7 ± 0.02 at 734 nm to obtain the test reagent. Then, 1.0 mL aqueous extracts of each sample with various concentrations (2.0, 1.5, 1.0, 0.5, and 0.1 mg/mL) were added to 2 mL of ABTS+ solution and mixed immediately as a test, while 1 mL of double-distilled water, which replaced the sample, was mixed with 2 mL of ABTS+ solution as the control. The double-distilled water was used as a blank solution. All samples were incubated in the dark at room temperature for 10 min; then, the absorbance was measured immediately at 734 nm using a UV-2250 spectrophotometer against a blank. Vitamin C was used as the reference standard. All tests were conducted in triplicate to calculate the mean values. The ABTS radical scavenging effect was calculated by the following formula:

Scavenging (%) = (A_control_ − A_test_)/A_control_ × 100

#### 4.3.4. Hydroxyl Radical Scavenging Activity Assay

The hydroxyl radical scavenging activity was assayed using the method from a previous study [42] with minor modifications. The hydroxyl radical was formed through a Fenton reaction in a system consisting of FeSO_4_ and H_2_O_2_. The mixture of 1.0 mL of 9 mM FeSO_4_, 1.0 mL of 9 mM salicylic acid, 1.0 mL of 8.8 mM H_2_O_2_, and 1.0 mL aqueous extract of each sample with various concentrations was incubated at 37 °C for 1 h as test absorbance. The absorbance was read at 510 nm with a UV-2250 spectrophotometer against a blank. Then, 1.0 mL of double-distilled water was used to replace the sample as the control absorbance and the reactant without hydrogen peroxide served as the sample absorbance. Vitamin C was used as the reference standard. All the tests were run in triplicate to calculate the mean values. The hydroxyl radical scavenging effect was calculated by the following formula:

Scavenging (%) = [1 − (A_test_ − A_sample_)]/A_control_ × 100

### 4.4. Assay of the Antioxidant Activities of the FDO and DDO In Vivo

#### 4.4.1. Test Animals

Male Kunming mice, weighing 20 ± 2 g, were obtained from the Experimental Animal Center of Anhui province, China. The mice were housed in plastic cages with a room temperature of 22 ± 2 °C, a suitable humidity of 60 ± 5%, and a cycle of 12:12 h light:dark. During the experiments, the mice were allowed free access to standard rat chow and tap water. All procedures of the animal experiments were carried out in accordance with the Ethics Committee of the Anhui University of Chinese Medicine.

#### 4.4.2. Carbon Tetrachloride (CCl_4_)-Induced Oxidative Toxicity

The mice were randomly divided into four groups with 12 mice in each group, after adapting to the environment for one week [43]. The normal control group and model group were treated with physiological saline solution for 10 days; the test groups were given FDO and DDO extracts with equivalent dry weight of 2 g/kg/d. All groups were given the solutions by gastric gavage. On the tenth day after administration, the mice in both the model group and the test groups fasted for 16 h and received an intraperitoneal injection of CCl_4_ (10 mL/kg b.w. of 10% CCl_4_ solution in olive oil). After 6 h following the animal model, the animals were weighed and killed. The normal control group was treated with an equal amount of olive oil in the same way.

#### 4.4.3. Determination of the Biochemical Parameters in the Liver and Serum

The blood and liver tissue samples were collected immediately, and the liver was washed with ice-cold saline. The blood samples were centrifuged at 4000× *g* at 4 °C for 10 min to obtain the serum. The liver was homogenized promptly with 0.1 g/mL ice-cold physiological saline to obtain the suspension, which was centrifuged at 1000 rpm for 10 min to collect the supernatant for further analysis. All the above-mentioned treatments were carried out at 4 °C.

The antioxidative parameters in the liver and serum including total antioxidant activity (T-AOC), glutathione (GSH), catalase (CAT), malondialdehyde (MDA), and the superoxide dismutase (SOD) level were determined using test kits, according to the manufacturer’s instructions, which were purchased from the Nanjing Jiancheng Bioengineering Institute in Jiangsu province, China.

#### 4.4.4. Histopathological Examination

After the mice were sacrificed, the liver tissue samples were instantly collected and fixed in 12% formalin. Then, the samples were dehydrated in graded (50–100%) alcohol and embedded in paraffin. Sections of 5 μm thick were obtained and stained with hematoxylin and eosin (H&E) for histopathological examination. All processes were based on the standard histology procedure and observed using a microscope at 200× magnification.

### 4.5. Extraction and Determination of the Polysaccharides in the FDO and DDO

The extraction of polysaccharides from the DO was conducted according to Chinese Pharmacopoeia (2015 version). The dried powders and fresh stems of DO were precisely weighed for equal amounts (dried weight as the counting unit) to gain the relevant polysaccharides of the DO with the following method. Briefly, 0.3 g DDO materials were extracted with 200 mL of deionized water and heating reflux for 2 h; then, they were cooled to room temperature, and deionized water was added to obtain a final volume of 250 mL. After filtration, 2 mL of the further filtrates were mixed with 10 mL anhydrous ethanol in a 15 mL centrifuge tube; then, they were refrigerated for 1 h. The mixtures were centrifuged at 4000 rpm for 20 min. After centrifugation, the supernatant was discarded; subsequently, the resulting precipitates were collected and washed with 8 mL aqueous 80% ethanol twice. Then, the precipitation was dissolved in hot water. The final polysaccharide sample was designated as the polysaccharide amount of the DDO.

The FDO was precisely weighed for an equal amount of DDO (dried weight as the counting unit) to obtain the relevant polysaccharide amount of the FDO with the following method. The FDO was squeezed into juice with 250 mL deionized water. According to the above-mentioned method, the same steps were carried out, including filtration, ethanol precipitation, centrifugation, washing, and dissolving. Finally, the polysaccharide sample of the FDO was obtained.

The content of total polysaccharides was determined by the phenol–sulfuric acid method [44]. Anhydrous glucose was used as a standard, and the total polysaccharide content of DO was expressed as the percentage of anhydrous glucose equivalents per 1 g of dried material. The polysaccharide content of the DO was determined by the ultraviolet spectrophotometer. The absorbance was read using a UV-2250 spectrophotometer at 488 nm against a blank containing all the reagents, except for the polysaccharide solution, which was replaced with double-distilled water.

### 4.6. Extraction and Determination of the Mannose in the FDO and DDO

The DO powdered stems were pre-extracted by aqueous 80% ethanol in a Soxhlet system for 4 h or until the elution was colorless, in order to remove pigments and impurities, respectively. The residues were decocted by 100 mL hot distilled water and 2 mL internal standard for 1 h; then, they were cooled to room temperature to obtain a final volume of 100 mL by mixing well. After centrifugation (2000 rpm, 10 min), 1 mL of the supernatant was collected in an ampoule, added to 0.5 mL of 3.0 M hydrochloric acid solution by mixing well, and sealed by hydrolysis at 110 °C for 1 h. Subsequently, it was cooled to room temperature and neutralized to PH 7.0 with 3 M aqueous NaOH. Briefly, each 0.4 mL of the mannose standard or the mannose of the hydrolyzed samples was mixed with a 0.5 M methanol solution of PMP (0.4 mL) and a 0.3 M aqueous NaOH (0.4 mL), respectively. Then, the mixture was kept in a water bath to react at 70 °C for 100 min. After cooling to room temperature, the mixture was added to 0.3 M hydrochloric acid solution (0.5 mL) and washed with 2 mL chloroform three times [45]. The chloroform was discarded, and the aqueous layer was filtered via a 0.22 μm membrane before HPLC analysis. Finally, 5 μL of each target sample was removed to inject into the HPLC system.

The content of mannose was determined by the HPLC (Agilent 1260) internal standard method, as follows. For the chromatography condition, a C 18 chromatographic column (4.6 mm × 250 mm, 5 μm) was used; for the acetonitrile, a 0.02 M solution of ammonium acetate (20:80) was used as a mobile phase, the flow rate was 1.0 mL/ min, the detection wavelength was 250 nm, and the column temperature was 30 °C. The mannose content was expressed as the percentage of mannose standard equivalents per 1 g of dried material. The extraction process was repeated three times for each sample. The final mannose content percentage was expressed by the mean value of the three measurements.

### 4.7. Determination of the Antioxidant Activities of the FDOP and DDOP In Vivo

All groups except for the test group were given 0.6 g/kg/d of FDOP and DDOP, respectively. All procedures and methods of this assay were carried using the experimental methods described in the Section 4.4.

### 4.8. Determination of the Molecular Weight of the FDOP and DDOP

The molecular weight of the FDOP and DDOP was measured by gel permeation chromatography (GPC) with an HPLC apparatus (Agilent 1260, USA), which was equipped with an ultrahydrogel column 1000 (300 mm × 7.8 mm) and an evaporative light scattering detector (ELSD) model 380. The specific operation conditions included: a mobile phase with ultrapure water; a flow rate of 1.0 mL/min; a column temperature of room temperature; an injection volume of 20 μL; and a running time of 15 min. To determine the molecular weight, the calibration curve was made using a series of Dextran T standards [46].

### 4.9. Monosaccharide Composition Analysis of the FDOP and DDOP

The monosaccharide composition of the FDOP and DDOP was analyzed by Gas Chromatography–Mass Spectrometry (GC-MS) [47]. Briefly, five milligrams of FDOP and DDOP were dissolved in 4 mL of 2 M trifluoroacetic acid solution (TFA), respectively, and were hydrolyzed at 100 °C for 12 h. The TFA in the hydrolysate was removed by repeatedly concentrating with methanol, which was transformed into alditol with NaBH_4_ and acetylated with acetic anhydride at 100 °C for 3 h. After being dried with anhydrous Na_2_SO_4_, the acetylated products were dissolved in chloroform and analyzed by the GC-MS apparatus, which was equipped with a Bruker SCION TQ GC-MS system, an EDR detector, and an HP-5 capillary column (30 m × 0.25 mm × 0.25 μm). The flow rate of the carrier gas (N2) was set at 1 mL/min. The column temperature was edited as follows: first, 150 °C for 1 min; then increased to 180 °C at 10 °C/min; and, finally, increased to 200 °C at 4 °C/min.

### 4.10. Statistical Analysis

All the measurements were performed in triplicate, and all the data are presented as the means ± standard deviations (S.D.s). The results, which contained the difference of antioxidant activity in vivo among samples, were analyzed using one-way analysis of variance (ANOVA) followed by the least significant differences (LSD) multiple comparison and Dunnett’s T3 tests with the SPSS statistical software version 17.0. Comparison between two group samples, such as the content of polysaccharide and mannose, was conducted with the independent sample *t*-test. In addition, *p*-values less than 0.05 (*p* < 0.05) were accepted as an indication of statistical significance.

## 5. Conclusions

Our work found that both FDO and DDO enhanced the antioxidant effects in vitro and in vivo, and the FDO was more effective than the DDO in increasing the antioxidant capacity. There were two main factors to illustrate the phenomena; one was that the content of polysaccharides in the FDO was higher than in the DDO, the other was that the monosaccharide compositions of the polysaccharides in the FDO were different from those in the DDO. It might also be possible that other chemical compositions led to this phenomenon, and it is worth further investigation in the future.

## Figures and Tables

**Table 1:** The content of Polysaccharide and Mannose in FDO and DDO.

Sample	Polysaccharide	Mannose
FDO	512.7 ± 13.32 **	489.5 ± 2.757 *
DDO	348.7 ± 12.82	291.3 ± 5.572

All results are presented as mean ± SD (*n* = 3) and analyzed by independent sample *t*-test. ** *p* < 0.01 compared with the DDO, * *p* < 0.05 compared with the DDO.

**Table 2:** Monosaccharide composition analysis of the FDOP and DDOP.

Polysaccharide Sample	Monosaccharide Composition (Molar Ratio)					
	Galactose	Glucose	Mannose	Aldose	Rhamnose	Xylose
FDOP	0.003	1.000	0.830	0.002	0.008	-
DDOP	0.0028	0.719	1.000	0.002	0.001	-

## Data Availability

The raw data supporting the conclusions of this manuscript will be made available by the authors, without undue reservation, to any qualified researcher.

## Sample Availability

Samples of the compounds are not available from authors.
